# Bolus Ingestion of Whey Protein Immediately Post-Exercise Does Not Influence Rehydration Compared to Energy-Matched Carbohydrate Ingestion

**DOI:** 10.3390/nu10060769

**Published:** 2018-06-14

**Authors:** Gethin H. Evans, Lewis Mattin, Isabelle Ireland, William Harrison, Adora M. W. Yau, Victoria McIver, Tristan Pocock, Elizabeth Sheader, Lewis J. James

**Affiliations:** 1 School of Healthcare Science, Manchester Metropolitan University, Manchester M1 5GD, UK; L.Mattin@mmu.ac.uk (L.M.); A.Yau@mmu.ac.uk (A.M.W.Y.); victoria.mciver@stu.mmu.ac.uk (V.M.); 2 Faculty of Biology, Medical and Health Sciences, University of Manchester, Manchester M13 9PT, UK; isabelle.ireland@student.manchester.ac.uk (I.I.); william.harrison@student.manchester.ac.uk (W.H.); Tristan.Pocock@manchester.ac.uk (T.P.); elizabeth.a.sheader@manchester.ac.uk (E.S.); 3 School of Sport, Exercise and Health Sciences, Loughborough University, Loughborough LE11 3TU, UK; L.James@lboro.ac.uk

**Keywords:** whey protein, maltodextrin, fluid balance, rehydration, gastric emptying, albumin, exercise, recovery

## Abstract

Whey protein is a commonly ingested nutritional supplement amongst athletes and regular exercisers; however, its role in post-exercise rehydration remains unclear. Eight healthy male and female participants completed two experimental trials involving the ingestion of 35 g of whey protein (WP) or maltodextrin (MD) at the onset of a rehydration period, followed by ingestion of water to a volume equivalent to 150% of the amount of body mass lost during exercise in the heat. The gastric emptying rates of the solutions were measured using ^13^C breath tests. Recovery was monitored for a further 3 h by the collection of blood and urine samples. The time taken to empty half of the initial solution (T_1/2_) was different between the trials (WP = 65.5 ± 11.4 min; MD = 56.7 ± 6.3 min; *p* = 0.05); however, there was no difference in cumulative urine volume throughout the recovery period (WP = 1306 ± 306 mL; MD = 1428 ± 443 mL; *p* = 0.314). Participants returned to net negative fluid balance 2 h after the recovery period with MD and 3 h with WP. The results of this study suggest that whey protein empties from the stomach at a slower rate than MD; however, this does not seem to exert any positive or negative effects on the maintenance of fluid balance in the post-exercise period.

## 1. Introduction

During exercise, particularly in hot environments, a degree of hypohydration is common, as sweat losses often exceed the intake of fluid [1]. Given that beginning exercise when hypohydrated appears to negatively influence endurance [2] and strength [3] performance, the restoration and maintenance of fluid balance after exercise is of importance, particularly when a further exercise bout is to be performed shortly afterwards.

Many studies have been carried out in the area of post-exercise rehydration, and these have determined that to restore fluid balance, the primary consideration should be the volume of fluid consumed, which needs to exceed that lost during exercise in order to account for ongoing urinary losses during the recovery period [4]. For the maintenance of fluid balance in the recovery period, the composition of an ingested solution is important. In particular, the addition of sodium, carbohydrate and protein to a solution has been shown to positively impact the maintenance of fluid balance after exercise-induced dehydration [5].

Protein is a commonly ingested substance following exercise, as it has been shown to positively influence post-exercise protein synthesis [6] and enhance the effectiveness of endurance training [7]. Whey protein is one of the most commonly consumed protein supplements; however, few studies have investigated the effect of this supplement on post-exercise rehydration. Seifert et al. [8] demonstrated that a 60 g/L carbohydrate drink +15 g/L of whey protein resulted in greater fluid retention than a 60 g/L carbohydrate drink only, when the same volume of fluid that was lost during exercise was replaced in a subsequent recovery period. Subsequently, James et al. [9] demonstrated that a solution containing 15 g/L of whey protein and 50 g/L carbohydrate resulted in no difference in fluid retention during recovery when compared to an energy-matched carbohydrate-only solution when a volume greater than the mass lost during exercise was ingested. Similar results have been observed when energy density has not been matched [10,11]. These investigations suggest that whey protein can be ingested without negatively influencing rehydration; however, they all involve the ingestion of a solution evenly over a one-hour period, i.e., the ingestion of a 20 g/L protein solution over a period of one hour. Protein, however, is commonly consumed, for recovery and remodelling purposes, immediately after exercise, with subsequent fluid ingestion in the form of water or sports drinks to help facilitate rehydration. Additionally, post-exercise bolus protein ingestion, at least, might augment muscle protein synthesis [12]. Therefore, the purpose of this study was to examine whether the ingestion of whey protein early in the recovery period followed by further ingestion of water results in different effects on fluid retention when compared to an energy-matched maltodextrin solution consumed in a similar fashion.

## 2. Materials and Methods

### 2.1. Participants

Eight (five males and three females) healthy volunteers completed this study (mean ± SD: age = 22 ± 3 years, height = 176 ± 8 cm, body mass = 72 ± 14 kg), which had prior ethical approval from the Faculty of Science and Engineering Faculty Ethics and the Research Governance Advisory Committee (Reference number: HCS201718_272). All participants completed a medical screening questionnaire and provided written informed consent prior to participation. Trials for females were not standardised based on their menstrual cycle phase, as previous work suggests these fluctuations do not affect post-exercise rehydration responses [13].

### 2.2. Experimental Protocol

Each participant completed two experimental trials with at least seven days between each trial. Experimental trials were completed in a randomised, counter-balanced order and began at the same time of the morning following an overnight fast from 9 p.m., except for the ingestion of 500 mL of water approximately one hour before arrival at the laboratory. In the 24 h prior to the first experimental trial, participants recorded their diet and physical activity and replicated these patterns prior to the second experimental trial.

Upon arrival at the laboratory, participants were instructed to provide a full-void urine sample before their body mass was measured (wearing minimal clothing) to the nearest 0.01 kg using a digital scale (Adam Equipment Co., Ltd., Milton Keynes, UK). Participants then began to exercise on a cycle ergometer in a chamber maintained at approximately 35 °C and 50% humidity. Participants undertook exercise, at an initial intensity of 2 W/kg body mass in blocks of 10 min interspersed with 5 min rest periods, during which time participants were weighed (in minimal clothing). Exercise continued at the same intensity unless participants were unable to continue working at this intensity at which point it was reduced until body mass was reduced by approximately 1.8% of the pre-exercise value. The same exercise protocol was followed in the subsequent trial. Participants left the heat chamber and showered, before being re-weighed (in minimal clothing) to determine the total amount of body mass lost during exercise. Participants provided another full-void urine sample before a blood sample (10 mL) was obtained from an antecubital vein via venepuncture.

Thirty minutes after the cessation of exercise, participants began the one-hour rehydration protocol, during which time a total fluid volume of 150% of body mass lost was provided. The initial bolus consisted of 500 mL of water with the addition of 35 g of whey protein (WP) or maltodextrin (MD). Both substrates were obtained from www.myprotein.com, and the WP contained a small amount of sodium (0.18 g), fat (2.66 g) and carbohydrate (1.4 g), whereas the MD did not. A batch analysis of solutions indicated osmolalities of 32 and 101 mosm/kg for MD and WP, respectively. Seventy-five milligrams of ^13^C-sodium acetate (Cambridge Isotope Laboratories Inc., Andover, MA, USA) was added to this bolus for assessment of the gastric emptying rate. The additional volume required to reach the 150% rehydration volume was divided into three aliquots of water provided at 15 min intervals (i.e., 15 min, 30 min and 45 min of the one-hour rehydration period). These contained no protein, maltodextrin or ^13^C-sodium acetate. Participants then rested in the laboratory for a further 3 h, during which time no further food or drink was consumed. Full-void urine samples were collected at hourly intervals, while 10 mL blood samples, via venepuncture, were collected after rehydration and at the end of the 3 h rehydration period. Movement was kept to a minimum throughout the recovery period.

### 2.3. Measurement of Gastric Emptying

Prior to ingestion of the first bolus of the rehydration solution, a basal end-expiratory breath sample was collected. Further end-expiratory breath samples were collected at 15 min intervals for 2 h after the ingestion of the first bolus of the rehydration solution. Breath samples were analysed using non-dispersive infra-red spectroscopy (IRIS Dynamic, Wagner Analysen-Technik, Bremen, Germany) to determine the ratio of ^13^CO_2_:^12^CO_2_. Differences in the ratio from baseline are expressed as delta over baseline (DOB) values. The half emptying time (T_1/2_) and time of maximal emptying (T_lag_) were calculated using the manufacturer’s integrated software.

### 2.4. Sample Handling and Analysis

For each 10 mL blood sample, 5 mL was collected into an EDTA tube for the measurement of haemoglobin and haematocrit, while the remaining 5 mL was collected into a serum separator tube which was centrifuged at 3000× *g* for 10 min at 4 °C. The resultant serum was stored at −80 °C. The Haemoglobin concentrations were assessed using a benchtop analyser (HemoCue, Angelholm, Sweden). Haematocrits were analysed via microcentrifugation. These values were used to calculate changes in blood, red cell, and plasma volumes relative to post-exercise values, using the equations of Dill and Costill [14]. Serum was analysed for osmolality via freezing point depression (Gonotec Osmomat 030 Cryoscopic Osmometer, Gonotec, Berlin, Germany) and for albumin concentration (Randox Daytona, Randox, Crumlin, UK). The total volume of urine at each time point was recorded and a sample was retained for the analysis of osmolality via freezing point depression, as described above. All samples were performed in duplicate, with the exception of haematocrit which was performed in triplicate.

### 2.5. Statistical Analysis and Calculations

Blood samples could not be collected from one participant, meaning all measures derived from blood samples are for seven participants instead of eight.

The net fluid balance was calculated relative to pre-exercise values, as participants were assumed to be euhydrated at this time point. The net fluid balance was determined at subsequent time points by subtracting the fluid lost through sweating (assuming all body mass lost through exercise was via sweating) and urine loss and adding the fluid gain through drinking where appropriate.

The free water clearance (FWC) was calculated using the equation

FWC = V(1 − U_osm_/S_osm_),

where V is the urine flow rate, U_osm_ is the urine osmolality and S_osm_ is the serum osmolality at a given time point.

Data were tested for normal distribution using the Shapiro–Wilk test and are presented as means ± SDs. Data containing two independent factors were analysed using a two-factor repeated measures analysis of variance (ANOVA). Violations for sphericity (as determined by Mauchly’s test of sphericity) were corrected via the Greenhouse–Geisser epsilon. Data containing one independent variable was analysed using a one factor repeated measures ANOVA or a dependent *t*-test with Bonferonni adjustments were appropriate. Analysis was undertaken using IBM SPSS version 23 and a critical value of 0.05 was utilised.

## 3. Results

### 3.1. Baseline Values, Exercise and Fluid Volume

The average pre-exercise body masses were 71.87 ± 13.76 kg and 71.83 ± 13.84 kg (*p* = 0.681) and the urine osmolalities were 487 ± 363 mosm/kg and 508 ± 344 mosm/kg (*p* = 0.804) for the MD and WP trials, respectively, indicating a consistent level of hydration status prior to undertaking exercise.

The exercise protocol was undertaken with similar environmental temperatures (MD = 35.5 ± 0.4 °C, WP = 35.2 ± 0.4 °C; *p* = 0.175) and humidities (MD = 52 ± 1%, WP = 52 ± 1%; *p* = 0.529) with a significant difference in exercise time (MD = 54 ± 7 min, WP = 58 ± 10 min; *p* = 0.048). Sweat loss (MD = 1384 ± 279 mL, WP = 1403 ± 308 mL; *p* = 0.449) and percentage body mass lost during exercise (MD = 1.92 ± 0.07%, WP = 1.95 ± 0.10%; *p* = 0.412) were, therefore, similar on both trials. Consequently, the total volume of fluid ingested during the rehydration period (MD = 2104 ± 455 mL, WP = 2094 ± 461 mL; *p* = 0.737) was similar for both trials.

### 3.2. Gastric Emptying Rate

**Figure 1:**
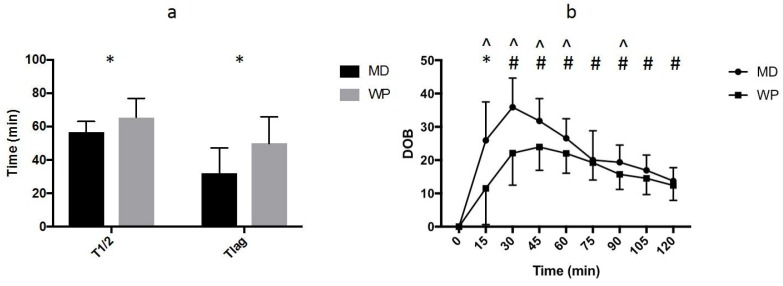
(**a**) Half emptying time (T_1/2_) and time of maximum emptying rate (T_lag_) (mins) during the maltodextrin (MD) and whey protein (WP) trials. “*” indicates a significant difference (*p* < 0.050) between trials. (**b**) DOB values during the maltodextrin (MD) and whey protein (WP) trials. “*” indicates significantly elevated time points (*p* < 0.05) from pre-ingestion for the MD trial, “#” indicates significantly elevated time points (*p* < 0.050) from pre-ingestion for the MD and WP trials and “^” indicates a significant difference (*p* < 0.050) between the MD and WP trials at a time point.

**Figure 2:**
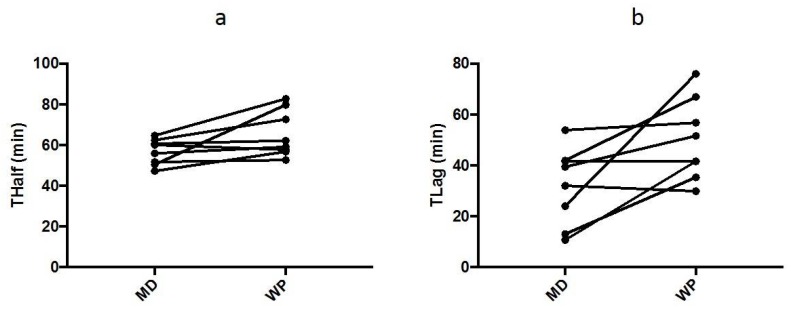
(**a**) Individual data for T_1/2_ and (**b**) T_lag_ during the maltodextrin (MD) and whey protein (WP) trials.

The T_1/2_ (Figure 1a) was slower (*p* = 0.050) during the WP trial (65.5 ± 11.4 min) compared to the MD trial (56.7 ± 6.3 min), and the T_lag_ (Figure 1a) was slower (*p* = 0.029) during the WP trial (50.0 ± 15.9 min) compared to the MD trial (32.1 ± 15.1 min). Individual data is shown in Figure 2. Main effects of trial (*p* = 0.005) and time (*p* < 0.001) and an interaction between them (*p* = 0.007) were observed for the DOB values. DOB values were elevated from pre-ingestion (*p* < 0.050) at all time points on the MD trial and from 30 min after the ingestion of WP. DOB values were lower (*p* < 0.050) on the WP trial at 15, 30, 45, 60 and 90 min after ingestion (Figure 1b).

### 3.3. Urine and Net Fluid Balance

**Figure 3:**
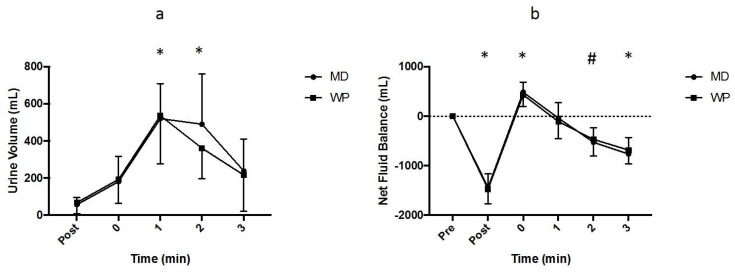
(**a**) Urine volume (mL) produced at each time point during the maltodextrin (MD) and whey protein (WP) trials. “*” indicates a significantly different time point (*p* < 0.050) from post-exercise value for the MD and WP trials. (**b**) The net fluid balance (mL) at each time point for the MD and WP trials. “*” indicates a significantly different time point (*p* < 0.050) from the pre-exercise value for the MD and WP trials, and “#” indicates significantly different time point (*p* < 0.050) from the pre-exercise value for the MD trial only.

A main effect of time (*p* = 0.001) was observed for the volume of urine produced during each trial (Figure 3a); however, no main effect of trial (*p* = 0.376) or an interaction between trial and time (*p* = 0.351) was observed. Compared to post-exercise, urine volume was increased (*p* < 0.050) at 1 and 2 h after rehydration for both the MD and WP trials. The total volume of urine produced (MD = 1428 ± 443 mL, WP = 1306 ± 306 mL; *p* = 0.314) after rehydration was, therefore, similar for both trials, as was the percentage of fluid retained (MD = 32.0 ± 15.1%, WP = 37.3 ± 13.3%; *p* = 0.342).

A main effect of time (*p* < 0.001) was observed for the net fluid balance (Figure 3b); however, no main effect of trial (*p* = 0.951) or interaction between time and trial (*p* = 0.362) was observed. The net fluid balance was reduced (*p* < 0.050) after exercise and increased (*p* < 0.050) after rehydration for both trials; however, participants returned to a negative fluid balance (*p* < 0.050) at 2 h after rehydration for the MD trial and at 3 h after rehydration for the WP trial.

A main effect of time (*p* = 0.070) was observed for urine osmolality; however, no main effect of trial (*p* = 0.243) or interaction between time and trial (*p* = 0.270) was observed. Post-hoc analyses were unable to detect any differences (*p* > 0.050) between time points.

### 3.4. Blood Analysis

**Figure 4:**
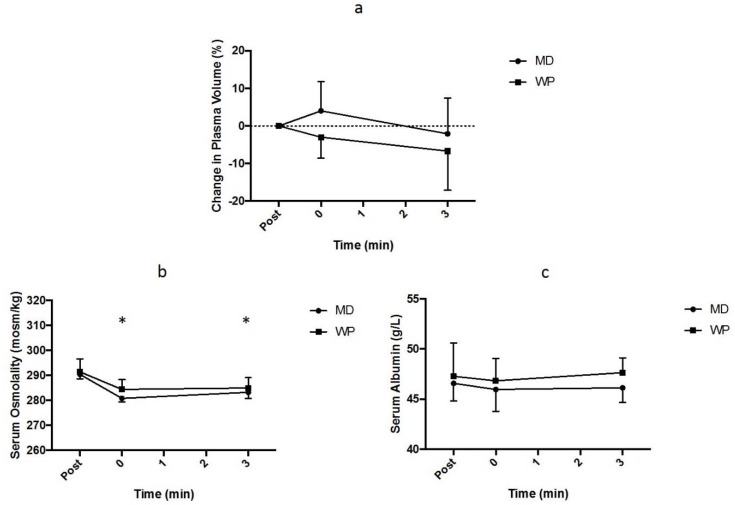
(**a**) Change in plasma volume (%) from post-exercise time points during the maltodextrin (MD) and whey protein (WP) trials. (**b**) The serum osmolality (mosm/kg) at each time point for the MD and WP trials. “*” indicates significantly difference (*p* < 0.050) from post-exercise time point for the MD and WP trials. (**c**) Serum albumin concentration (g/L) at each time point during the MD and WP trials.

No main effects of time, trial or an interaction between time and trial (*p* > 0.050) were observed for the percentage change in blood, red cell or plasma volume (Figure 4a).

A main effect of time (*p* < 0.001), but no main effect of trial (*p* = 0.095) or interaction between trial and time (*p* = 0.357), was observed for serum osmolality (Figure 4b). The serum osmolality was reduced (*p* < 0.050) from post-exercise values immediately after, and 3 h after, rehydration for both trials.

No main effect of time (*p* = 0.702) or interaction between trial and time (*p* = 0.728) occurred, but a main effect of trial (*p* = 0.038) was observed for serum albumin (Figure 4c). Post-hoc analyses were unable to detect further differences between trials; however, serum albumin tended to be higher (*p* = 0.086) during the WP trial compared to the MD trial at 3 h after rehydration.

### 3.5. Free Water Clearance

No main effects of trial (*p* = 0.474), time (*p* = 0.326) or an interaction between trial and time (*p* = 0.154) were observed for free water clearance.

## 4. Discussion

The main finding of this study was that there was no difference in fluid retention following exercise-induced dehydration amounting to approximately 2% of body mass between whey protein and maltodextrin when these substrates were ingested at the start of a rehydration period and followed by water ingestion. Whey protein appeared to empty from the stomach at a slower rate than maltodextrin; however, this did not lead any differences in blood or urine markers related to whole body fluid balance when the total fluid volume over a 1 h period was equivalent to 150% of body mass lost.

Previous studies have demonstrated that a volume of solution greater than the volume of fluid lost in sweat is required to ensure a return to positive fluid balance [4]; however, numerous studies have demonstrated that participants end a sustained recovery period in negative fluid balance. In this study, despite ingesting a total fluid volume greater than that lost through sweat, participants had net fluid balances at 3 h after completing rehydration of −764 ± 332 mL and −684 ± 283 mL for the MD and WP trials, respectively. Previous studies have demonstrated that the addition of carbohydrates [15,16] and protein [8] to a rehydration solution may be more beneficial than water for maintaining fluid balance. Despite this, neither of these substrates blunted the drinking-induced diuresis sufficiently to prevent a return to negative fluid balance within 2–3 h of drinking, at least in the concentrations tested. In contrast, the addition of sodium to a rehydration solution seems to have a more pronounced effect on fluid balance mechanisms, and, when ingested in high enough quantities, may be sufficient to ensure that fluid balance is maintained for up to 6 h after rehydration [17,18]. The reason for these observations may be related to the mechanisms by which carbohydrates, protein and sodium influence the post-exercise fluid balance.

A number of studies have investigated the effect of protein, or protein-containing solutions on post-exercise rehydration. The ingestion of milk protein, either in the form of semi-skimmed milk or in a milk protein isolate solution appears to be beneficial for rehydration when compared to commercially available carbohydrate-electrolyte solutions [19,20,21]. However, the effects of whey protein, a commonly consumed nutritional supplement amongst athletes and regular exercisers, are less consistent and unclear. Seifert et al. [8] examined the effects of a 60 g/L carbohydrate, 15 g/L whey protein solution on post-exercise rehydration, compared to a 60 g/L carbohydrate solution and water when the total fluid volume ingested during rehydration was matched to sweat losses during exercise. It was reported that the addition of whey protein, in this study, enhanced fluid retention. In contrast, James et al. [10] demonstrated that the addition of 20 g/L of whey protein to a 60 g/L carbohydrate solution did not influence fluid retention when a fluid volume greater than that lost during exercise was ingested. Subsequent studies supported the observation that whey protein has little effect on rehydration when compared to carbohydrates when total fluid intake volumes are greater than sweat losses, either when energy density is matched [9] or not [11], at least when the rehydration solution is ingested rapidly (i.e., within 1 h) after exercise. The present study supports these observations as there was no difference in fluid retention between trials when energy density was matched and the fluid volume ingested was greater than sweat losses. One of the novel aspects of this study is the manner in which the substrate of interest was delivered. Previous studies investigating the effects of protein on post-exercise rehydration have provided the substrate evenly over a designated period [8,9,10,11,20,21] which is not necessarily representative of how athletes and regular exercisers ingest nutritional supplements such as whey protein.

In this study, 35 g of whey protein or maltodextrin was provided in 500 mL of water at the onset of rehydration with only water being provided following this to give a total fluid volume of 150% of body mass lost. The results suggest that the timing/manner of substrate ingestion does not seem to influence subsequent fluid balance mechanisms as participants returned to negative fluid balance at similar times to other studies. Interestingly, Li et al. [22] reported increased fluid retention for solutions containing 22 g/L or 33 g/L whey protein, compared to a carbohydrate-electrolyte solution, when a volume equivalent to 150% of sweat lost was consumed over a more protracted time period (150 min). Thus, whilst the present study suggests the timing of substrate intake might not matter, the ingestion rate of a whey protein-containing rehydration solution might be an important consideration for its efficacy. An additional consideration for this and previous studies is that food was not consumed in the post-exercise recovery period. It is unlikely that an athlete would not eat in the 4 h post-exercise period, and usually, this food intake would contain a mixture of macronutrients that might influence rehydration. A previous study [23] demonstrated that the addition of sodium to a rehydration solution still enhanced post-exercise rehydration, even when a standardised meal was consumed during the rehydration period. However, sodium and protein/macronutrients likely act via separate mechanisms to influence rehydration, and future studies should aim to examine the impact of eating typical recovery meals alongside various rehydration strategies to determine their combined success.

The mechanisms by which protein may influence post-exercise rehydration are not well understood. There are two potential mechanisms by which protein may effect fluid balance mechanisms when a fluid volume greater than that lost during exercise is ingested. Firstly, protein-containing solutions may empty from the stomach at different rates to other solutions and/or be absorbed at different rates within the intestine. Indeed, even different protein sources might differentially influence absorption rates [24]. These effects on delivery may then influence the extent of dilution of the plasma volume and change in serum osmolality that could affect arginine vasopressin release [25] and urine output. Secondly, the ingestion of certain nutrients may influence the osmotic pressure and the extent of fluid that is retained once it is present in the circulation.

The addition of carbohydrates to a rehydration solution has been shown to reduce the gastric emptying rate [26,27] and this, in part, contributes to the reduced urine output observed following the ingestion of such solutions as it slows the delivery of water to the circulation. This prevents haemodilution and ultimately, urine production [15,16]. The addition of protein to a solution should, in theory, reduce the gastric emptying rate as solutions empty from the stomach in a linear fashion that is related to energy density [28]; however, this has not previously been measured in a post-exercise rehydration study. In the present study, WP emptied from the stomach at a slower rate compared to an energy-matched MD solution when assessed via ^13^C breath testing. This did not, however, correspond to any differences in the plasma volume or serum osmolality response, suggesting that the delay in gastric emptying that occurred following the ingestion of 35 g of whey protein was not sufficient to affect fluid balance mechanisms.

Albumin is the main protein present in plasma and, as such, has a large effect on the oncotic pressure and plasma volume [29]. Li et al. [22], reported an elevated plasma albumin content following the ingestion of whey protein-containing rehydration solutions, along with enhanced fluid retention, suggesting an increase in plasma albumin content might, at least partially, explain the enhanced rehydration. However, in the present study, whilst the plasma albumin concentration tended to be higher 3 hours after the rehydration period following WP compared to MD, there was no interaction effect and there was no influence on plasma volume or urine output.

WP contained a small amount of sodium. Sodium is well known to enhance post-exercise rehydration [17,18,23], but sodium concentrations less than ~25–30 mmol/L do not significantly alter post-exercise rehydration. Therefore, the small amount of sodium consumed in the WP trial, which amounted to <6 mmol/L is unlikely to have influenced the rehydration responses in this study.

## 5. Conclusions

In conclusion, this study demonstrated that the ingestion of 35 g of whey protein at the onset of a rehydration period, followed by the ingestion of water to give a total fluid volume equivalent to 150% of the body mass lost during exercise did not benefit or inhibit fluid retention during a subsequent recovery period, compared to 35 g of maltodextrin.
